# Mechanically Induced Switching Between Orbital‐ and Fano‐Resonance Rectification in a Dual‐Anchored Molecular Junction

**DOI:** 10.1002/anie.3716518

**Published:** 2026-04-30

**Authors:** Xin Sun, Ran Liu, Samjhana Maharjan, Sneha Kindapal, Guang Yang, Feng Sun, Chuan‐Kui Wang, A. Jean‐Luc Ayitou, Bingqian Xu

**Affiliations:** ^1^ Single Molecule Study Laboratory College of Engineering and Nanoscale Science and Engineering Center University of Georgia Athens Georgia USA; ^2^ Biodesign Center for Bioelectronics and Biosensors Arizona State University Tempe Arizona USA; ^3^ Department of Chemistry University of Illinois Chicago Chicago Illinois USA; ^4^ School of Physics Ningxia University Yinchuan P.R. China; ^5^ Key Laboratory of Medical Physics and Image Processing of Shandong Province School of Physics and Electronics Shandong Normal University Jinan P.R. China

**Keywords:** molecular junctions, molecular rectification, molecular switching, molecular‐metal interference

## Abstract

Achieving precise control over charge transport through individual molecules is central to advancing single‐molecule electronics. In short molecular junctions can exhibit rectification from fundamentally different mechanisms, yet strong sensitivity to contact geometry and electrode‐molecule coupling often obscures whether diode behavior arises from asymmetric orbital alignment or quantum interference. Here, we demonstrate dual‐mode rectification in a mechanically addressable metal‐molecule‐metal junction by chemically programming the interface with a heterofunctional scaffold bearing thiol and carboxyl anchors. Using scanning tunneling microscopy break‐junction (STM‐BJ) measurements under controlled mechanical modulation, we observe two reproducible conductance states that are most consistently assigned to two contact configurations on the basis of converging mechanical, statistical, and theoretical evidence. Current–voltage analysis further shows that the state assigned to the S–Au/COO–Au (thiolate‐carboxylate) configuration rectifies through asymmetric molecular‐orbital alignment and electrode coupling, whereas the state assigned to the nominally symmetric COO–Au/COO–Au (carboxylate–carboxylate) configuration rectifies via an interference‐driven, bias‐dependent Fano‐resonance pathway. These findings demonstrate that anchored chemical synthons, combined with mechanical control of binding geometry, provide a practical strategy for engineering and directly comparing rectification mechanisms in short single‐molecule junctions.

## Graphical abstract

Mechanical control of anchoring chemistry enables switching between orbital‐driven and interference‐driven rectification in a single‐molecule junction.

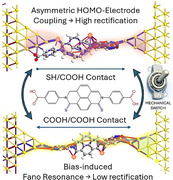

## 1 Introduction

Molecular rectification and reliable conductance switching in metal‐molecule‐metal junctions are key functionalities for molecular‐scale optoelectronics (e.g., photoswitches) [1, 2, 3, 4], electromechanics (e.g., strain sensors) [5, 6, 7, 8], and bioelectronics (e.g., DNA sequencing) [9, 10, 11]. Various stimuli have been used to modulate single‐molecule charge transport, with light and electrochemical stimuli/triggers being the most widely studied [12, 13, 14]. Examples typically operate through photoinduced isomerization or cyclization reactions [4, 15], and electrochemical redox reactions [16, 17, 18], rectification, in contrast, is often governed by more subtle symmetry breaking at the electrode–molecule interfaces and its evolution under bias, making mechanistic assignment and reproducibility particularly challenging[19]. At the single‐molecule level, rectification is typically rationalized within two broad mechanistic frameworks. In an orbital‐controlled mechanism, rectification arises from unequal level alignment and electrode coupling, such that the dominant frontier orbital enters the bias window asymmetrically and produces polarity‐dependent transmission [20, 21, 22]. In an interference‐controlled mechanism, rectification can be amplified when transport is shaped by quantum interference, including Fano resonances generated by coupling between molecular states of different spatial character, such as localized and more delocalized transport channels [17, 23]. Because both mechanisms are exquisitely sensitive to contact chemistry and binding geometry, even small interfacial variations can reshape the transmission line shape and bias dependence, complicating interpretation across nominally similar junctions.

Beyond light and electrochemical stimuli, mechanical modulation has emerged as an attractive alternative, enabling junction properties tuning without altering a molecule's chemical structure [24]. Previous studies have shown that compressing or stretching a molecular junction can induce pronounced changes in conductance, primarily through changes in contact geometry and molecule‐metal interactions [25, 26, 27]. For example, pyridyl‐terminated molecular wires exhibit conductance switching due to multiple coordination geometries at the Au‐N interface [28]. However, for small molecules, reliable mechanical modulation remains challenging, because specific conformations are transient in break‐junction experiments and the electrode–molecule interfaces are difficult to control precisely [29, 30, 31]. These limitations make it difficult to use mechanical modulation as a clean probe of rectification mechanisms within a single molecular platform. They also highlight the need for systems in which the interfacial structure is chemically programmed, while mechanical motion simply selects between a small number of well‐defined configurations.

Anchoring groups provide a direct chemical handle over interfacial structure, energy‐level alignment, and electrode coupling [32, 33]. Thiol (–SH) and amine (–NH_2_) groups are the most widely used in single‐molecule studies because they form quasi‐covalent bonds with metallic electrodes, ensuring robust electrical contacts [34, 35]. Thiols, in particular, have high binding affinity to gold (Au─S bond dissociation energy ≈ 2 eV versus ≈ 0.15 eV for Au–NH_2_ [36, 37], but they are prone to oxidation, chemical degradation, and competitive displacement under ambient or electrochemical conditions [38, 39, 40], and also could have physisorbed bonding in deposition of dithiols [41]. Amines form weaker bonds but yield narrower conductance distributions and more predictable binding geometries [32, 42], providing a useful contrast in how interfacial chemistry influences coupling and transport symmetry.

To isolate the role of anchor groups in governing the interfacial chemistry of molecular junctions, carboxyl (–COOH) anchors offer an alternative contact chemistry. Compared with thiol (–SH) and amine (–NH_2_) anchors, carboxyl groups exhibit tunable binding characteristics, enhanced chemical stability, and diverse coordination modes—monodentate, bidentate, and bridging when attached to gold through oxygen coordination [34, 36, 42, 43]. These properties offer greater flexibility in tuning energy‐level alignment and coupling. However, Au–OOC junctions remain relatively underexplored in the context of mechanically controlled experiments, partly because their weaker Au–O interactions hinder reproducible junction formation, and their multiple coordination configurations introduce additional challenges for precise charge transport control [32, 35, 44]. At the same time, carboxylate groups are not always innocent, under specific electrochemical conditions, they can display redox activity and even decarboxylative reactivity [45, 46]. Although electrochemical studies have shown that Au–OOC interactions can be tuned by adjusting the electrode surface charge [47], their behavior under purely mechanical modulation and the extent to which carboxyl anchoring can be used to select between different rectification mechanisms have not been systematically examined.

**FIGURE 1:**
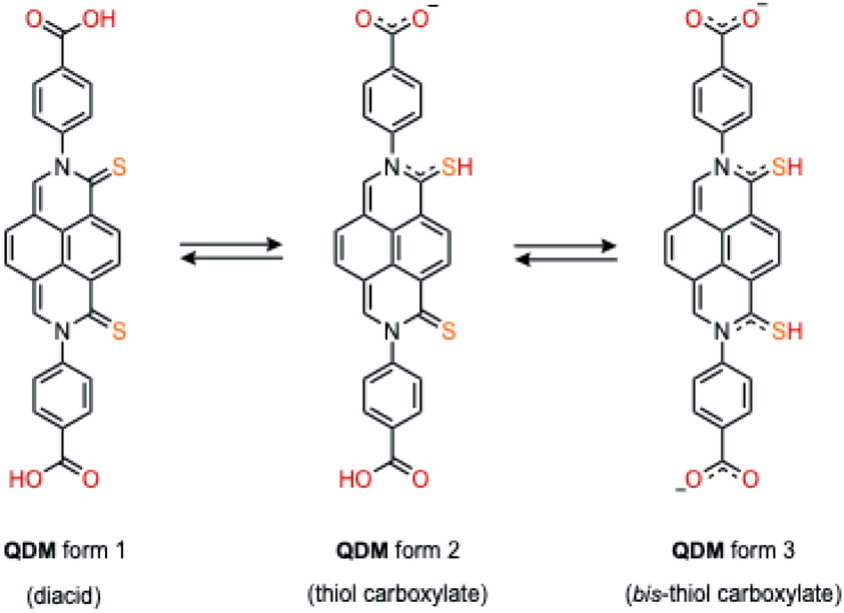
Different plausible chemical form 1 (diacid), form 2 (thiol carboxylate), and form 3 (bis‐thiol carboxylate) of napthoquinodimethyl‐bis‐thioamide bis‐carboxylic acid (QDM‐diacid).

In this work, we address this gap by chemically programming the interface to realize dual‐mode rectification within a mechanically addressable single‐molecule junction. We design a heterofunctional QDM‐diacid molecule that bears competing –SH and –COOH anchors, creating two reproducible states assigned to a S–Au/COO–Au (thiolate–carboxylate) configuration and a COO–Au/COO–Au (carboxylate–carboxylate) configuration with comparable binding strengths (Figure 1), based on converging evidence from conductance and displacement statistics, mechanical interconversion, pulling‐rate dependence, and DFT/transmission calculations. The QDM‐diacid backbone is aromatic and highly conjugated, providing a chemically robust scaffold for probing interfacial transport through distinct anchoring motifs [48]. We systematically investigate a largely unexplored system based on this QDM‐diacid scaffold—the controllable mechanical modulation of a single heterofunctional molecule that can be switched between two distinct contact configurations associated with its –SH and –COOH functional groups, where the competition between configurations arises from the comparable binding strengths of the two anchor groups [47, 49]. Using the scanning tunneling microscopy break‐junction (STM‐BJ) technique [50], we leverage reversible configurations and thereby switch between two rectifying states with well‐defined conductance levels. By correlating electrode displacement with conductance states, we reveal how binding chemistry, molecular orbital resonance, and electrode coupling select the charge transport mechanism. We further identify an optimal modulation amplitude range for reproducible rectification switching and attribute the distinct rectification behaviors of the two configurations to asymmetric orbital alignment and Fano resonance. Overall, this study demonstrates a simple yet powerful strategy for achieving conductance switching through mechanical modulation, harnessing the distinct electronic characteristics of different anchor chemistries to advance the design of functional nanoscale electronic devices. Together, these results demonstrate that anchoring chemistry, combined with mechanical modulation of binding geometry, provides a practical strategy for engineering molecular rectifiers with mechanically selectable functional states.

## 2 Results and Discussion

**FIGURE 2:**
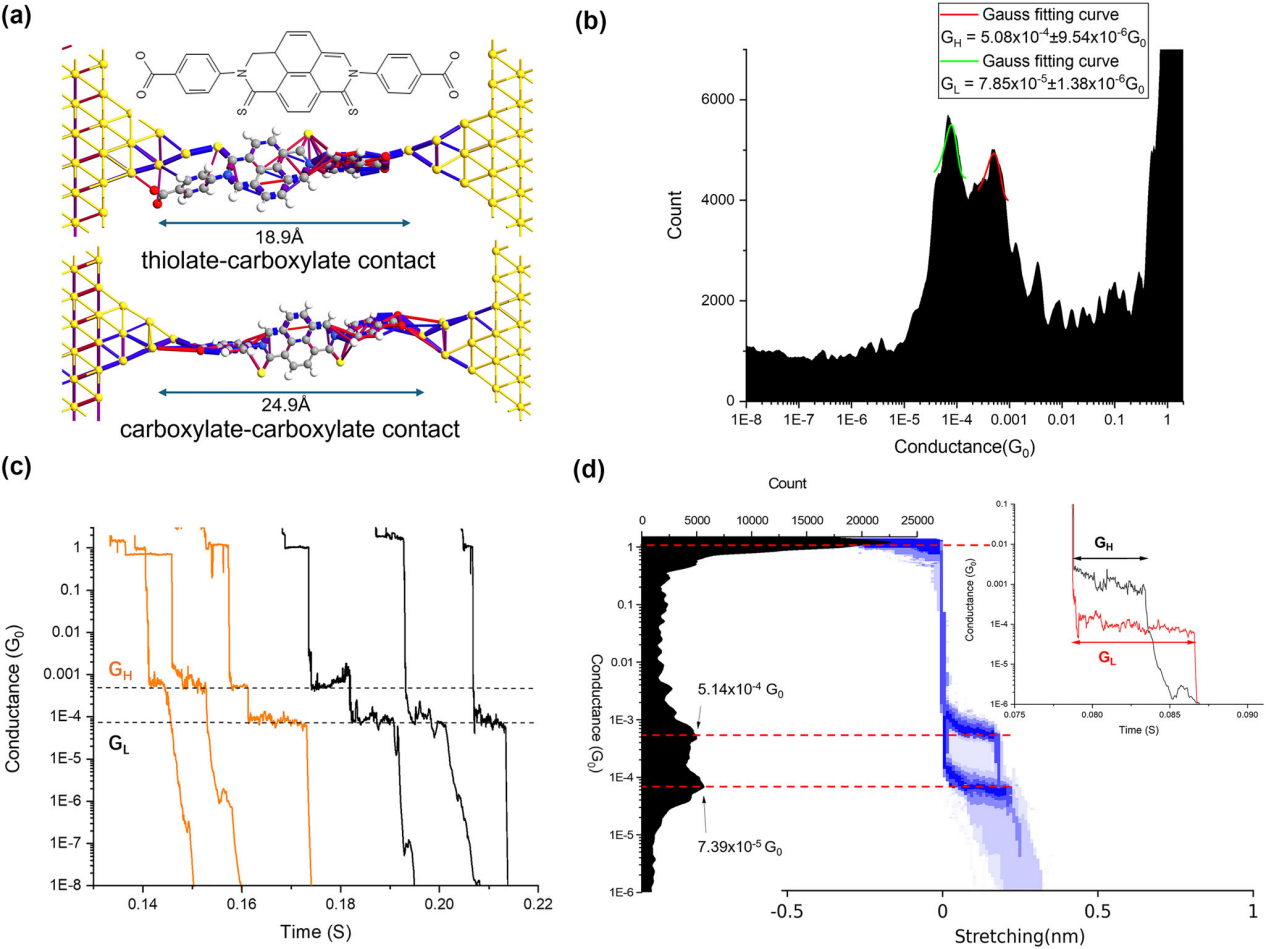
Junction formation and characterization: (a) Chemical structure of the QDM‐diacid and schematic illustrations of the two proposed junction configurations, S–Au/COO–Au (thiolate‐carboxylate) and COO–Au/COO–Au (carboxylate‐carboxylate). The blue horizontal lines beneath the molecular models indicate the corresponding equilibrium junction lengths. (b) One‐dimensional (1D) conductance histogram for QDM‐diacid, compiled from thousands of break‐junction cycles, showing two reproducible high (G_H_) and low (G_L_) conductance peaks. (c) Representative conductance‐displacement traces showing G_H_ and G_L_ conductance plateaus. (d) Two‐dimensional (2D) conductance‐displacement histogram (blue) statistically compiled from thousands of continue stretching traces. The dense plateaus in the 2D histogram occur at the same conductance values as the two peaks in the 1D histogram (black). The inset highlights representative traces used to compare the plateau lengths of the two states, illustrating that the G_L_ state has a longer plateau than the G_H_ state.

We employed the STM–BJ technique to fabricate and characterize single‐molecule junctions [50, 51]. In this approach, a gold tip is repeatedly brought into contact with, and then retracted from, a gold substrate functionalized with a monolayer of the target molecule. During retraction, a constant bias (200 mV) is applied, and the current is recorded. Stable molecular bridges give rise to conductance plateaus in the current–displacement traces (Figure 2c). Thousands of traces are collected to construct conductance histograms, where peaks correspond to the most probable conductance values (Figure 2b). Additional details are available in Supporting Information (SI‐3).

To probe the effect of anchoring chemistry on junction geometry and mechanical response, we synthesized napthoquinodimethyl‐bis‐thioamide bis‐carboxylic acid (QDM‐diacid) (Figure 1 and SI‐2 Figures S1–S7). This heterofunctional molecule incorporates two distinct anchoring sites: a thiol (–SH) group laterally positioned on the molecular backbone (blue in Figure 2a) and a terminal carboxyl (–COOH) group (red in Figure 2a), supported by the calculations (Figure S9). The 1D conductance histogram (Figure 2b) reveals two well‐separated and reproducible conductance peaks, denoted state G_L_ (7.39 × 10^−5^ G_0_) and G_H_ (5.14 × 10^−4^ G_0_), suggesting the presence of two accessible junction configurations. Combined with the following experimental analysis and theoretical calculations, these two states are most consistently assigned to a symmetric carboxylate–carboxylate configuration and a mixed thiolate‐carboxylate configuration, respectively. Notably, the G_L_ state is more frequently observed, within the assignment framework, this trend is consistent with the oxygen‐coordinated junction being more readily formed under our measurement conditions. Plateau‐length analysis (Figure 2d, inset) (SI‐4, Figure S9) shows that G_L_ junctions persist slightly longer than G_H_ junctions, consistent with coordination bonding at the electrode–molecule interface rather than weaker van der Waals contacts [52, 53]. Given that the measurements were performed in air under ambient, non‐electrochemical conditions, and the conductance states are reproducible and reversibly accessible, we do not attribute this behavior to irreversible chemical decomposition such as oxidative decarboxylation.

**FIGURE 3:**
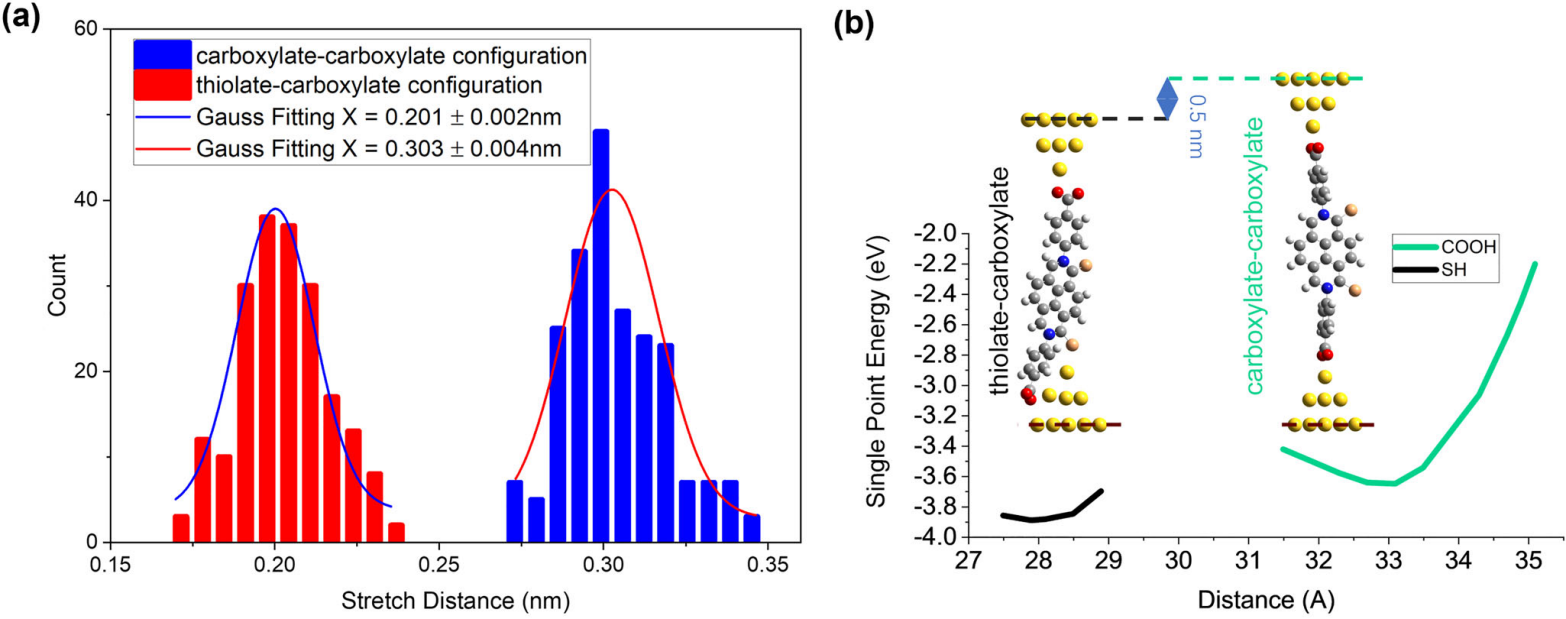
Mechanical stability and transitions: (a) Histograms of plateau retraction distance for the G_H_ (red) and G_L_ (blue) states, with Gaussian fits. (b) Calculated single‐point energies versus junction elongation for the two configurations. The calculated elongation difference is consistent with the experimentally observed difference in plateau length between the two conductance states.

We next examined junction stability under elongation by constructing displacement‐distance (Δd) histograms for both configurations (Figure 3a). The G_L_ state displays a longer mean plateau length than G_H_, indicating greater variability in junction geometry. This difference suggests a possible elongation pathway in which some G_H_ junctions transition to the more extended G_L_ configuration before rupture, consistent with traces in Figure 2c that show G_H_ → G_L_ transitions. The 2D conductance‐displacement histogram (Figure 2d) reveals an initial short decay region (∼0.06 nm) preceding each plateau, representing a transition from through‐space tunneling to through‐molecule transport [54]. Despite differences in the anchor chemistry, the two junctions have comparable total break lengths. This can be explained by a trade‐off between geometry and binding strength: the carboxylate–carboxylate junction is intrinsically longer but less strongly bound, whereas the thiolate‐carboxylate junction is shorter but more strongly bound to electrode. This is consistent with the differing elastic stress curves observed at these two molecular junctions (see Figure S9). Calculated equilibrium junction elongation lengths differ by ∼0.15 nm (see SI‐4), in good agreement with the experimental plateau length difference (∼0.10 nm) (Figure 3b), suggesting that both factors contribute to mechanical stability and supporting the proposed geometrical ordering of the two states.

**FIGURE 4:**
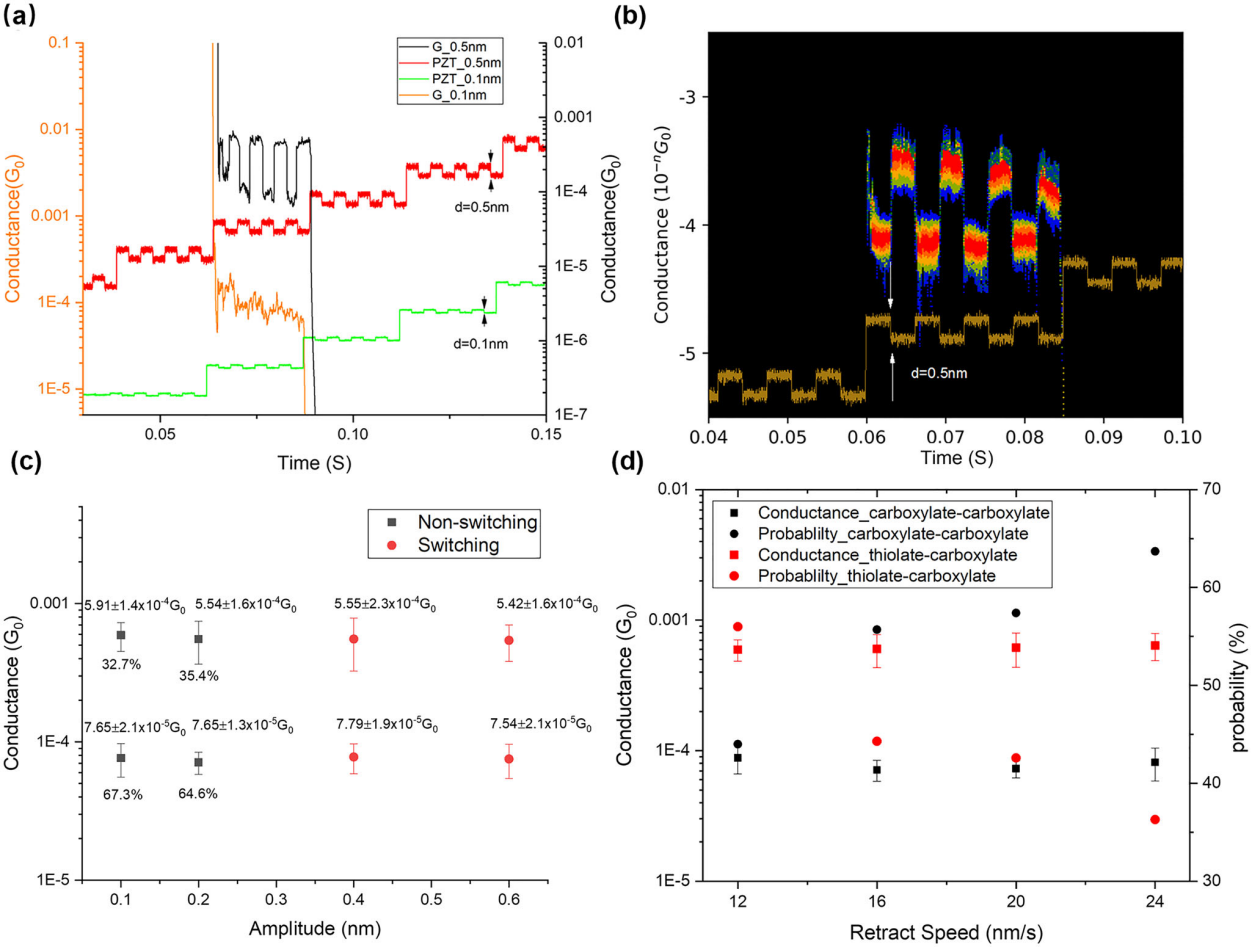
Active modulation and kinetics: (a) Conductance traces during piezo modulation at amplitudes d = 0.5 nm and 0.1 nm, showing the presence and absence of reversible switching between the two conductance states. (b) 2D histogram showing reversible G_H_⇆G_L_ switching during piezo modulation. (c) Formation probability and mean conductance of each state versus modulation amplitude. (d) Formation probability as a function of tip retraction speed, showing the kinetic preference toward the carboxylate‐carboxylate state at higher retract speed.

To actively drive junction switching, we applied piezo‐modulation during STM–BJ measurements [8, 55, 56], as detailed in the Supporting Information (SI‐3). After forming a molecular junction, the tip–substrate distance was modulated at 135 Hz with varying amplitudes (d = 0.1–0.8 nm). Clear, reversible switching between G_H_ and G_L_ states was observed for amplitudes of 0.4–0.6 nm (Figure 4a,b), matching the calculated difference in optimized junction length between the two configurations (∼0.5 nm) (Figures 2a and 3b). At smaller amplitudes (0.1–0.3 nm), junctions remained in their initial state; at d ≥ 0.7 nm, junction rupture dominated.

Formation‐probability analysis was performed by classifying molecular traces into the G_H_ and G_L_ states based on their conductance plateau, detailed procedures are provided in the Supporting Information (SI‐3). As shown in Figure 4c, carboxylate‐carboxylate junction are preferred over all three amplitudes, in agreement with static histogram data (Figure 2b). Retraction‐speed experiments (Figure 4d) further uncover a kinetic bias: increasing retraction rate enhances the carboxylate–carboxylate formation probability from 44% at 12 nm/s to 63.7% at 24 nm/s, indicating that rapid elongation dynamically traps the system in the extended carboxylate–carboxylate geometry before relaxation into the more compact thiolate‐carboxylate configuration state.

The restricted modulation amplitude window for reversible G_H_/G_L_ switching, together with the retraction speed‐dependent probability shift, indicates that G_H_ and G_L_ are mechanically related junction states, not randomly unrelated motifs. Within the assignment proposed here, these dynamic observations are consistent with interconversion between a shorter thiolate‐carboxylate junction and a more extended carboxylate‐carboxylate junction.

**FIGURE 5:**
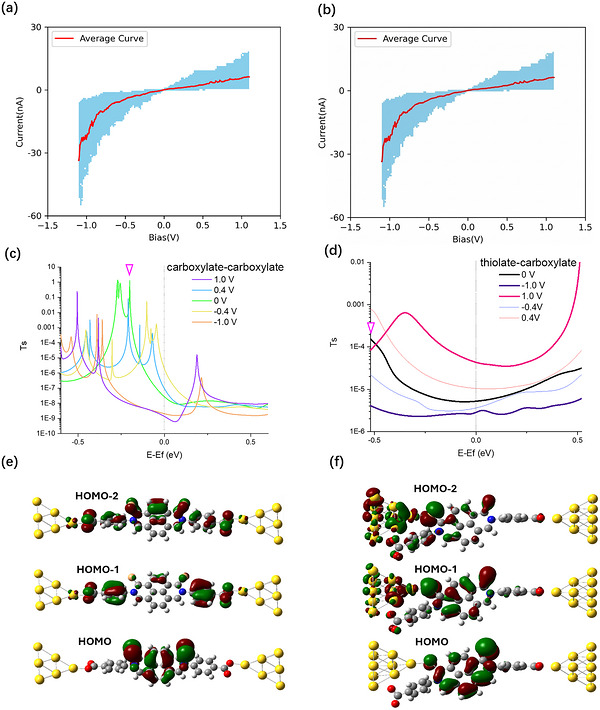
Electronic transport mechanisms: (a, b) 2D histograms and representative average *I*–*V* curves for the COO–Au/COO–Au a) and S–Au/COO–Au (b) configurations. (c, d) Calculated transmission spectra *T*(*E*, *V*) plotted as a function of electron energy related to the Fermi level under different applied bias for the corresponding configurations. (e, f) Representative MPSH orbitals for the occupied states most relevant to charge transport property in the carboxylate–carboxylate (e) and thiolate‐carboxylate (f) junctions, respectively, illustrating the different orbital characteristics associated with the two rectification mechanisms.

To compare electron transport properties, *I*–*V* characteristics were acquired for each stable junction [10, 57]. After identification of a conductance plateau, retraction was paused and the bias was swept from −1.1 V to + 1.1 V (SI‐3, Figure S8). Only stable traces were included in the final 2D *I*–*V* histograms (Figure 5a,b). Both junctions exhibit pronounced rectification, with sharply enhanced current under negative bias; notably, the thiolate‐carboxylate junction shows a larger rectification ratio (9.8) than the carboxylate–carboxylate junction (4.3).

To elucidate the origin of rectification, density functional theory (DFT) and non‐equilibrium Green's function (NEGF) calculations were performed using QuantumATK^65^. Currents were calculated within the Landauer–Büttiker framework [58],

$$I=e/h\int T\left(E,V\right)\left[f\left(E-\mu_{L}\right)-f\left(E-\mu_{R}\right)\right]dE$$

where 𝑇(E, V) = 𝑇𝑟[Γ_𝐿_𝐺^𝑟^Γ_𝑅_𝐺^𝑎^]. 𝑓 is the Fermi‐Dirac distribution function of electrodes, 𝐺^𝑟(𝑎)^ and Γ_𝐿(R)_ denote the Green's functions and electrode–molecule couplings, respectively. As discussed previously [59], asymmetric coupling shifts molecular orbitals by different amounts under bias. The rectification of the thiolate‐carboxylate junction is attributed to its intrinsic asymmetric junction geometry and electrode coupling. This interpretation is further supported by the dI/dV measurements in Figure S8c, which show a pronounced conductance enhancement under negative bias. As shown in the calculated transmission spectrum (Figure 5d), as the bias voltage sweeps, the Highest Occupied Molecular Orbital (HOMO) gradually migrates into the bias window with the bias voltage, leading to strong bias‐dependent level alignment under negative bias (Figure 5f) and a monotonic reduction of the HOMO–LUMO gap with increasing negative voltage. The electron distribution of the molecular orbitals demonstrates that the HOMO is primarily localized on the S‐Au contact, leading to much stronger coupling with one electrode. Consequently, the HOMO's energy level is strongly modulated by the applied bias in negative direction but not the other. This peak position aligns with the transmission peak, supporting the interpretation that rectification in the thiolate‐carboxylate junction arises from asymmetric HOMO alignment and unequal electrode coupling.

In contrast, the carboxylate–carboxylate junction exhibits different rectification behavior associated with a Fano resonance arising from interference between localized and delocalized occupied molecular states [60]. In the calculated transmission spectra (Figure 5c), this resonance appears as a broadened dip‐like interference feature rather than an ideal sharp Fano line shape. To understand this, the evolution of the molecular orbitals within the bias window is calculated (Figure 5e). For the carboxylate–carboxylate junction, the orbitals respond asymmetrically to the bias field. Under negative bias, a set of nearly degenerate molecular orbitals split non‐uniformly. This results in the formation of a new localized orbital while others remain delocalized and coupled to the electrodes. The continued localized (HOMO) state concentrated on the central QDM backbone, and delocalized (HOMO‐1, HOMO‐2) background state distributed over the conjugated molecular backbone, satisfies the Fano resonance conditions [61]. Owing to appreciable molecule‐electrode coupling, this Fano resonance appears in the calculated transmission as a broadened dip‐like interference feature rather than an ideal sharp peak‐dip line shape. As this interference feature evolves with bias, transmission is suppressed more strongly under one polarity than the other, leading to asymmetric conductance and rectification (Figures 5c,e and SI‐4 Figure S10).

## 3 Conclusion

In summary, we identified two reproducible and mechanically interconvertible conductance states in this QDM‐diacid junction. Based on converging evidence from conductance/displacement statistics, reversible mechanical switching, pulling rate‐dependent state populations, and DFT/transmission calculations, these states are most consistently assigned to a more extended COO–Au/COO–Au (carboxylate–carboxylate) configuration and a shorter S–Au/COO–Au (thiolate‐carboxylate) configuration. Within this assignment framework, the two states exhibit two well‐defined and reversibly accessible rectifying conductance states whose interconversion is controlled by mechanical modulation. We further show that junction formation is kinetically governed: increasing the retraction speed preferentially stabilizes the extended, low‐conductance carboxylate–carboxylate configuration by outpacing the relaxation required to form the thiolate–carboxylate configuration. *I*–*V* analysis reveals that this nominally symmetric carboxylate–carboxylate junction nonetheless displays rectification (ratio = 4.3) via an interference‐driven, bias‐dependent Fano resonance, a mechanism fundamentally distinct from the asymmetric HOMO alignment and electrode coupling responsible for the higher rectification (ratio = 9.8) in the thiolate‐carboxylate junction, manifested in the transmission spectrum as a broadened dip‐like feature. Together, these results identify carboxyl‐terminated scaffolds and dual‐anchor architectures as a versatile platform for mechanically responsive molecular rectifiers and highlight pulling dynamics as a key parameter for selecting anchoring geometry and charge transport mechanism. More broadly, they establish a chemically programmable, kinetically selectable strategy for tailoring charge‐transport behavior in single‐molecule devices through coordinated control of anchoring chemistry, binding geometry, and quantum interference.

## Author Contributions

**Xin Sun**: data curation, writing – review and editing, writing – original draft, methodology, formal analysis. **Ran Liu**: software, data curation, methodology, writing – review and editing. **Samjhana Maharjan**: methodology, data curation. **Sneha Kindapal**: data curation, formal analysis. **Guang Yang**: data curation, formal analysis. **Feng Sun**: data curation, methodology. **Chuan‐Kui Wang**: data curation, formal analysis, methodology. **A. Jean‐Luc Ayitou**: investigation, funding acquisition, formal analysis, data curation, supervision, methodology. **Bingqian Xu**: conceptualization, investigation, methodology, formal analysis, data curation, funding acquisition, writing – original draft, writing – review and editing, validation.

## Conflicts of Interest

The authors declare no conflicts of interest.

## Supporting information



The authors have cited additional references within the Supporting Information [13, 51, 58, 61, 62, 63].
**Supporting File**: anie72443‐sup‐0001‐SuppMat.pdf.

## Data Availability

The data that support the findings of this study are available from the corresponding author upon reasonable request.
