# Supplementary material for: Mechanically Induced Switching Between Orbital‐ and Fano‐Resonance Rectification in a Dual‐Anchored Molecular Junction
Source: Angew Chem Int Ed Engl. 2026 Apr 30;65(25):e3716518. doi: 10.1002/anie.3716518 (PMC13266942; doi:10.1002/anie.3716518)
Supplement: Supplementary file 1 — The authors have cited additional references within the Supporting Information [13, 51, 58, 61, 62, 63]. Supporting File: anie72443‐sup‐0001‐SuppMat.pdf. [file ANIE-65-e3716518-s001.pdf]

Supplementary Information (15 pages)

Mechanically Induced Switching between Orbital- and Fano-Resonance Rectification in a Dual-Anchored Molecular Junction

Xin Sun[a], Ran Liu*[a] [e], Samjhana Maharjan[b], Sneha Kindapal[a], Guang Yang[b], Feng Sun[c], Chuan-Kui Wang[d], A. Jean-Luc Ayitou*[b], Bingqian Xu*[a]

[a] Single Molecule Study Laboratory, College of Engineering and Nanoscale Science and Engineering Center, University of Georgia, Athens, GA 30602, USA.
[b] Department of Chemistry, University of Illinois Chicago, Chicago, IL 60607, USA
[c] School of Physics, Ningxia University, Yinchuan 750021, P.R. China
[d] Key Laboratory of Medical Physics and Image Processing of Shandong Province, School of Physics and Electronics, Shandong Normal University, Jinan 250358, P.R. China
[e] Biodesign Center for Bioelectronics and Biosensors, Arizona State University, 1001 S. McAllister Ave. Tempe, AZ 85287

Correspondence: Bingqian Xu (nanoxu@uga.edu) | A. Jean-Luc Ayitou (aayitou@uic.edu) | Ran Liu (ran.liu.6@asu.edu);

This document includes:

Supplementary Text (SI 1-4)

Supplementary Figure S1-10

Related References 1-5

Table of Content

SI-1. Chemicals and Materials…………………………………………………………......................... 2

SI-2. Synthetic procedures & characterizations of QDM-diacid molecule…………………….…… 2

SI-3. STMBJ IV measurements …………………………………..…………………..……..……10

SI-4. Theoretical calculations……………………..……... 12

References.…………………………..……... 15

SI-1. CHEMICAL AND MATERIALS

Preparation of QDM-diacid molecules sample and STM setup

The dissolved QDM-diacid molecules by DMSO solution (EMD Millipore Corporation) were dropped on a freshly hydrogen flame annealed Au (111) substrate for 20 min incubation, followed by a thorough washing with water and ethanol alternatively. The substrates were prepared by thermally evaporating gold onto freshly cleaved mica (Electron Microscopy Sciences, Muscovite Mica sheet V1 Quality). Then the sample was kept in a chamber and the break junction measurement was immediately performed to prevent contamination. The single molecule conductance measurement was performed using a STM break junction platform developed previously in our lab[1]. QDM-diacid molecular junctions are created by repeatedly engage and withdraw a cut gold tip (Alfa Aesar gold wire, 0.25mm dia, Premion 99.999%) with the fresh gold substrate as electrode.

SI-2. SYNTHETIC PROCEDURES & CHARACTERIZATION

Synthesis of QDM-diacid

QDM-diacid used in the experiments was synthesized by the following steps[2, 3]:

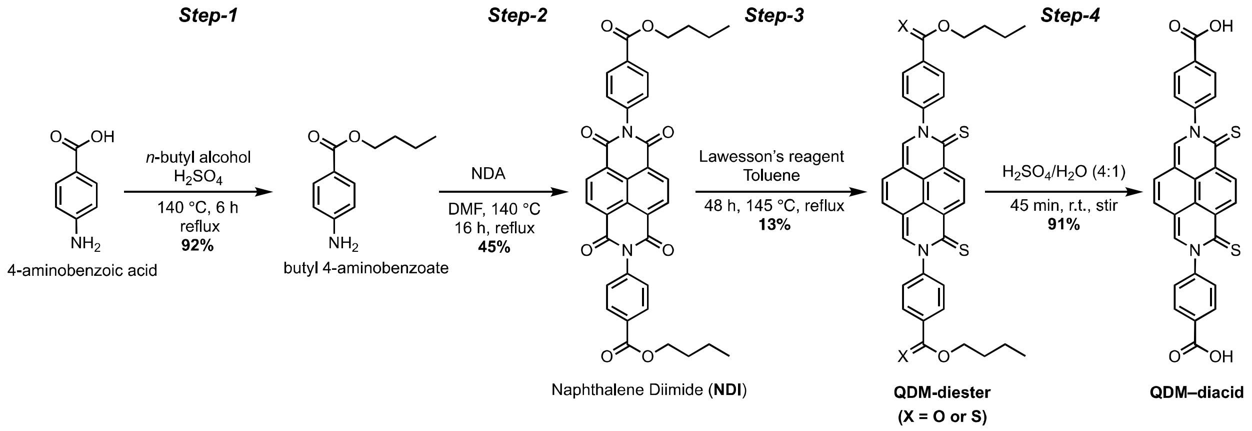

Figure S1. Synthetic route for QDM–diacid.

Step-1: Formation of butyl 4-aminobenzoate

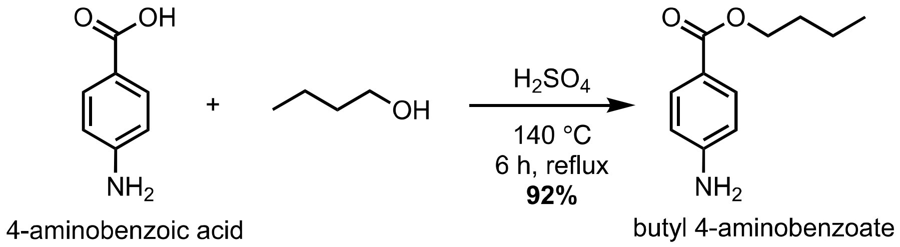

4-aminobenzoic acid (5.0 g, 36.5 mmol, 1 equiv.) was dissolved in n-butyl alcohol (50 mL) in a 250 mL round-bottom flask. Concentrated sulfuric acid (6.0 mL) was added dropwise to the reaction mixture. The mixture was heated to reflux at 140°C and stirred continuously. The progress of the reaction was monitored using thin-layer chromatography (TLC). Upon completion, the reaction mixture was cooled to room temperature and carefully quenched by the addition of a saturated sodium bicarbonate solution. The resulting neutral solution was extracted with ethyl acetate (3 x 150 mL). The combined organic layers were dried over anhydrous sodium sulfate (Na_2SO_4), filtered, and concentrated under reduced pressure. The product was obtained as an off-white solid (6.49 g, 92% yield).

^{1}H NMR (300 MHz, Chloroform-*d*) δ = 7.85 (d, *J* = 8.7 Hz, 2H), 6.64 (d, *J* = 8.5 Hz, 2H), 4.26 (t, *J* = 6.6 Hz, 2H), 4.05 (br, 2H), 1.72 (m, *J* = 6.8 Hz, 2H), 1.47 (m, *J* = 7.1 Hz, 2H), 0.97 (t, *J* = 7.3 Hz, 3H) ppm.

Step-2: Formation of **NDI**

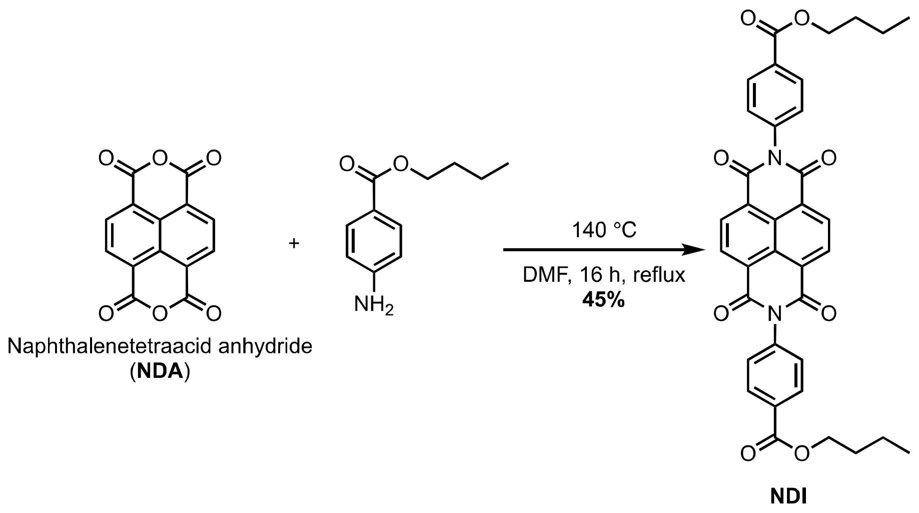

Naphthalenetetracarboxylic (3.0 g, 11.2 mmol, 1 equiv.) was dissolved in anhydrous dimethylformamide (60 mL) in a 250 mL round-bottom flask. Butyl 4-aminobenzoate (6.49 g, 33.6 mmol, 3 equiv.) was then added to the solution. The reaction mixture was heated to reflux at 140°C, with continuous stirring. The progress of the reaction was monitored by thin-layer chromatography (TLC). After completion, the reaction mixture was allowed to cool to room temperature and then filtered. The solid residue was collected, dried, and analyzed by NMR spectroscopy. The final product was obtained as a solid (3.1 g, 45% yield).

^{1}H NMR (300 MHz, Chloroform-*d*) δ = 8.87 (s, 4H), 8.27 (d, *J* = 8.6 Hz, 4H), 7.44 (d, *J* = 8.6 Hz, 4H), 4.39 (t, *J* = 6.6 Hz, 4H), 1.79 (m, *J* = 6.5 Hz, 4H), 1.51 (q, *J* = 7.5 Hz, 4H), 1.00 (t, *J* = 7.4 Hz, 6H) ppm.

Step-3: Formation of **QDM-diester**

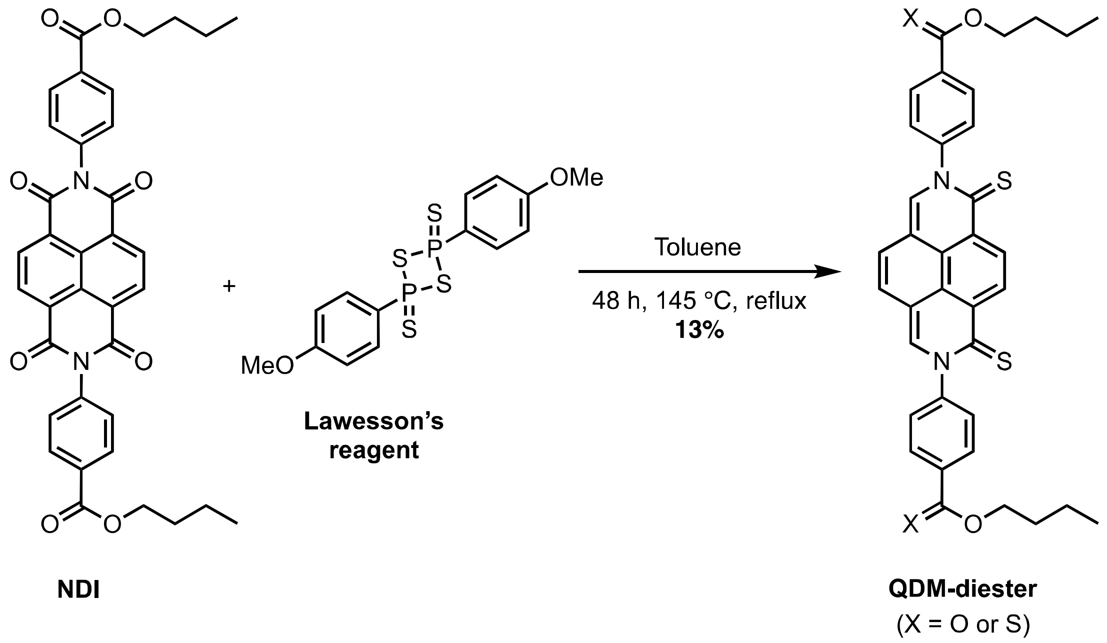

NDI (3.1 g, 5.01 mmol, 1 equiv.) was dissolved in anhydrous toluene (50 mL) in a suitable reaction vessel. Lawesson's reagent (9.81 g, 24.26 mmol, 5 equiv.) was added to the solution. The reaction mixture was heated to reflux at 145°C and stirred continuously for 48 hours.

After the reaction was complete, the mixture was concentrated under reduced pressure to remove the solvent. The resulting crude product was washed twice with methanol to remove impurities. The crude material was then purified by column chromatography, using a gradient of 2% acetone in toluene as the eluent. The desired product was obtained as a solid (419 mg, 13% yield).

^{1}H NMR (500 MHz, Chloroform-*d*) δ = 9.17 (s, 2H), 8.41 (d, J = 8.4 Hz, 4H), 7.78 (s, 1H), 7.49 (d, J = 8.1 Hz, 4H), 7.03 (s, 2H), 4.72 (t, J = 6.4 Hz, 4H), 1.94 (m, J = 6.7 Hz, 4H), 1.57 (q, J = 7.6 Hz, 8H), 1.04 (t, J = 7.4 Hz, 6H) ppm.

^{13}C NMR (125 MHz, Chloroform-*d*) δ = 182.1, 165.6, 149.4, 135.0, 132.0, 131.5, 131.0, 127.3, 126.1, 123.5, 118.9, 117.3, 65.5, 30.9, 29.9, 19.4, 13.9 ppm.

Step-4: Formation of **QDM-diacid**

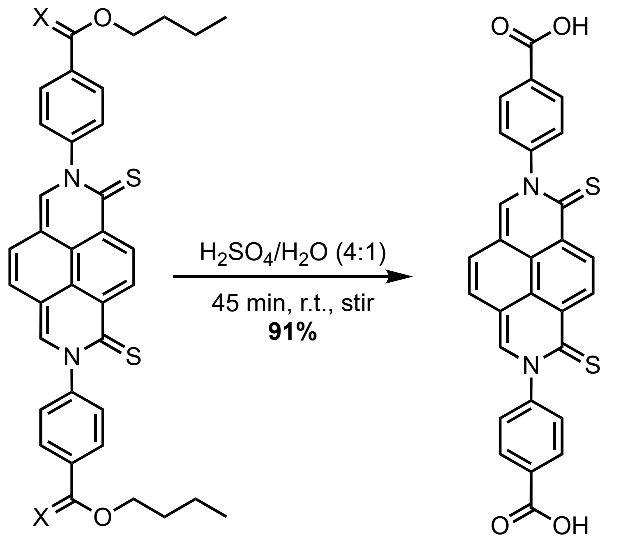

QDM-diester
(X = O or S)

QDM–diacid

QDM-diester (419 mg, 0.675 mmol) was placed in a 50 mL round-bottom flask. A mixture of concentrated sulfuric acid and water (H_2SO_4: H_2O = 4:1) was prepared, and 5 mL of this solution was added dropwise to the reaction mixture at 0°C. The acid mixture was added in 1 mL portions with constant stirring while maintaining the temperature in an ice bath.

After the addition was complete, the reaction mixture was allowed to warm to room temperature and stirred for 45 minutes. The resulting precipitate was collected by filtration and washed several times with water to remove residual acid. The product was obtained as a dark wine-red solid (313 mg, 91% yield).

^{1}H NMR (500 MHz, Chloroform-*d*) δ = 8.96 (s, 2H), 8.51 (s, 2H), 8.15 (d, *J* = 8.6 Hz, 5H), 7.68 (d, *J* = 8.6 Hz, 4H), 7.30 (s, 2H) ppm.

^{13}C NMR (125 MHz, Chloroform-*d*) δ = 206.9, 180.1, 167.0, 149.6, 134.7, 133.9, 131.6, 131.1, 130.8, 128.1, 126.4, 123.8, 118.8 ppm.

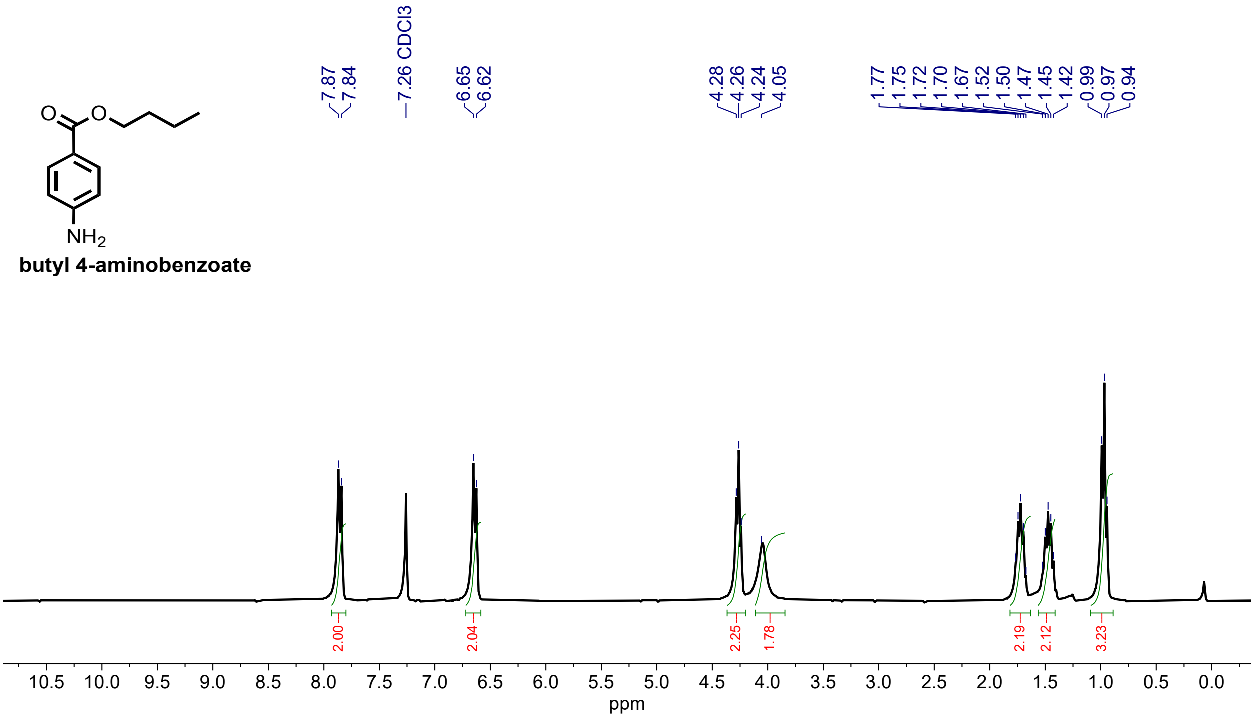

Figure S2. ^{1}H NMR (300 MHz) of butyl 4-aminobenzoate in Chloroform-*d*.

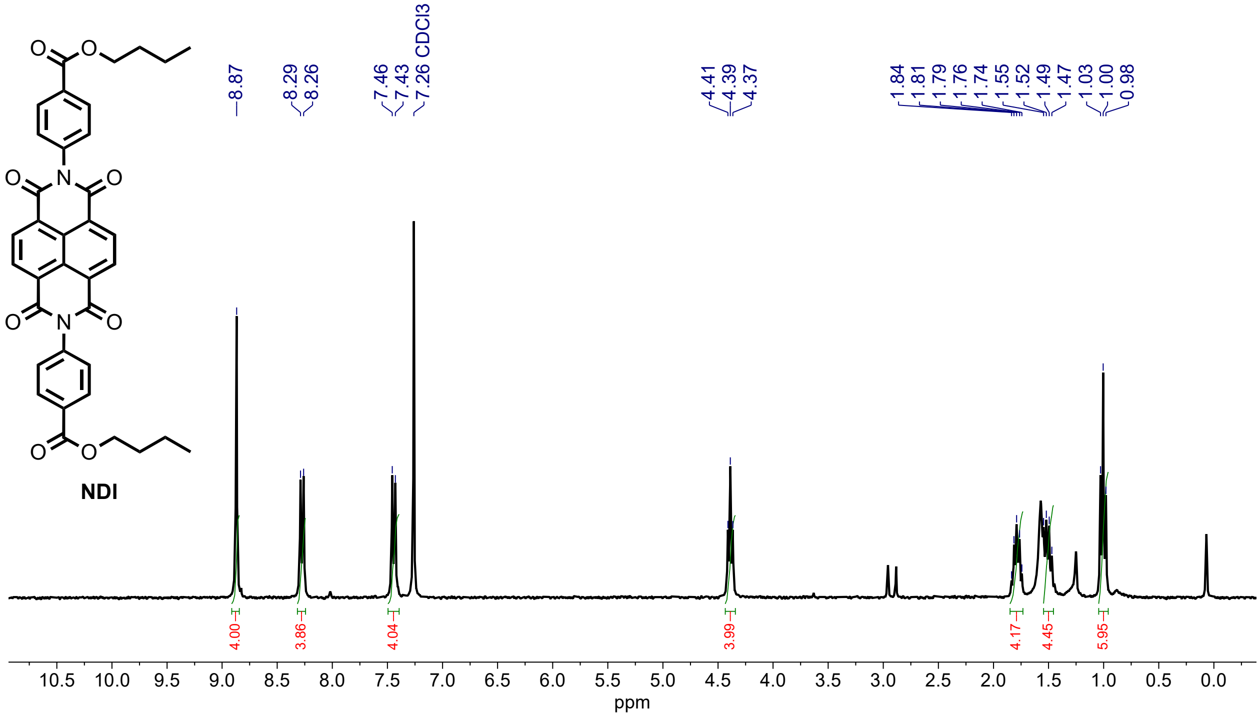

Figure S3. ^{1}H NMR (300 MHz) of **NDI** in Chloroform-*d*.

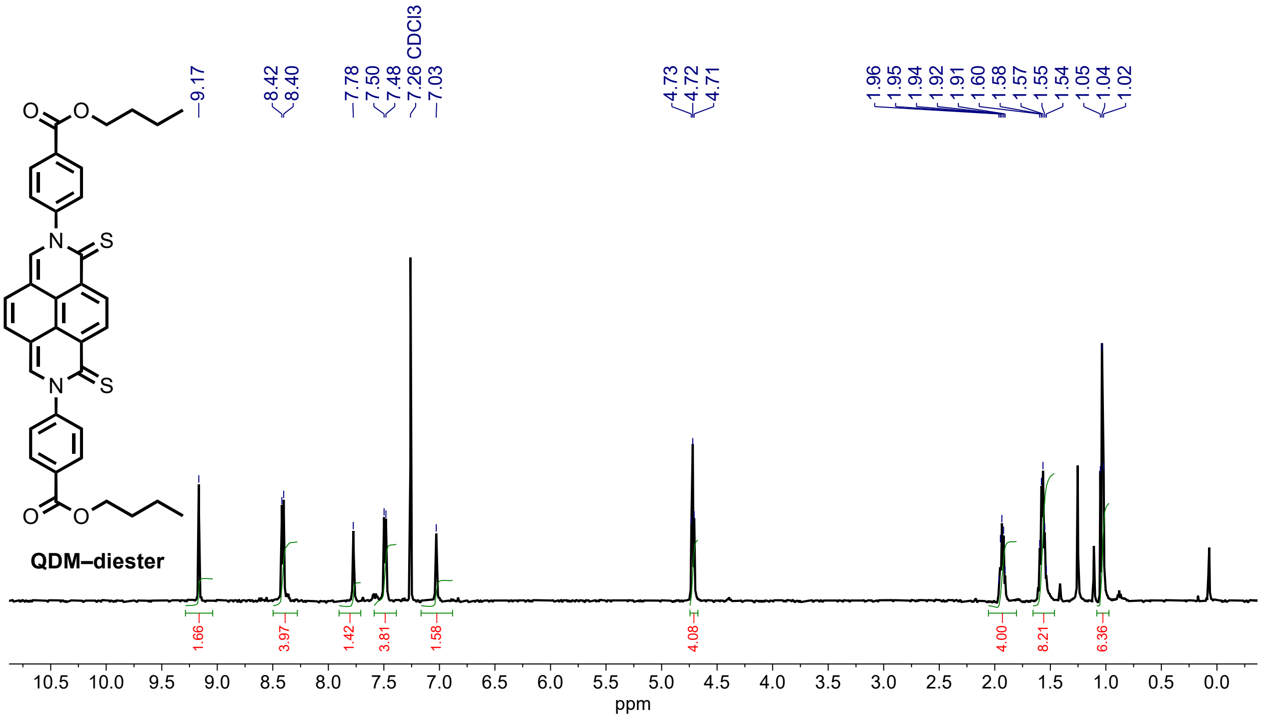

Figure S4. ^{1}H NMR (500 MHz) of QDM–diester in Chloroform-*d*.

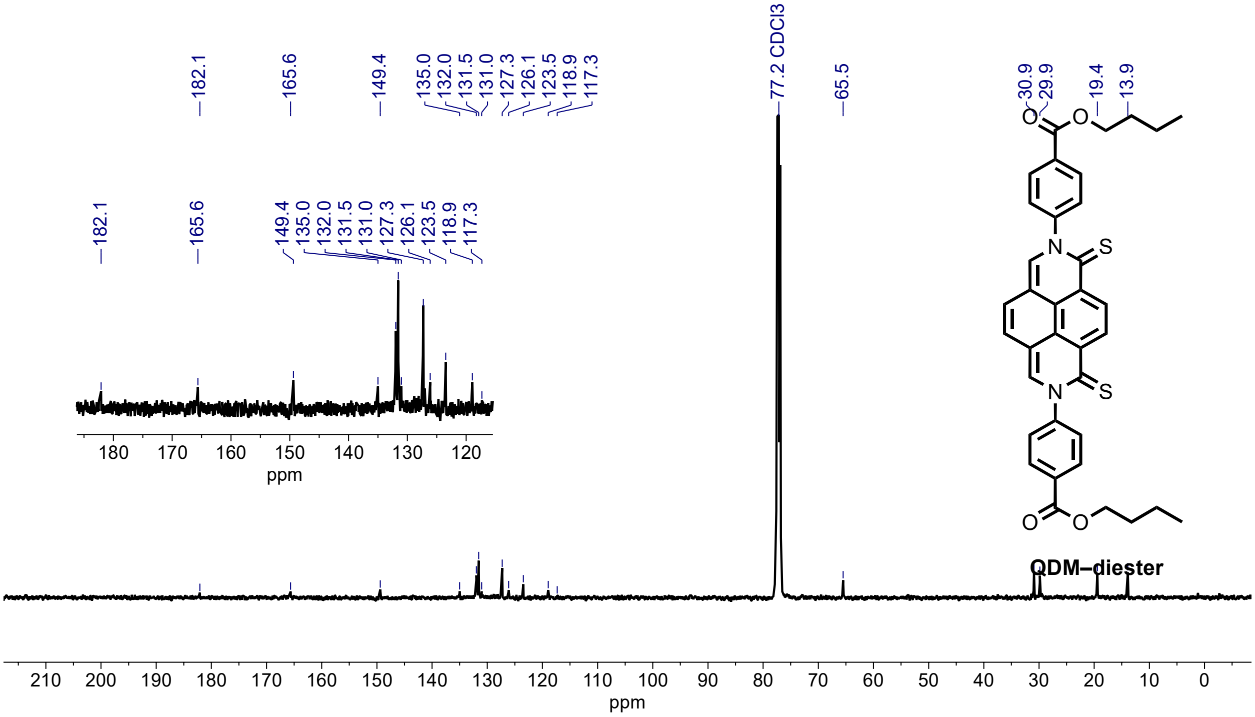

Figure S5. ^{13}C NMR (125 MHz) of QDM–diester in Chloroform-*d*.

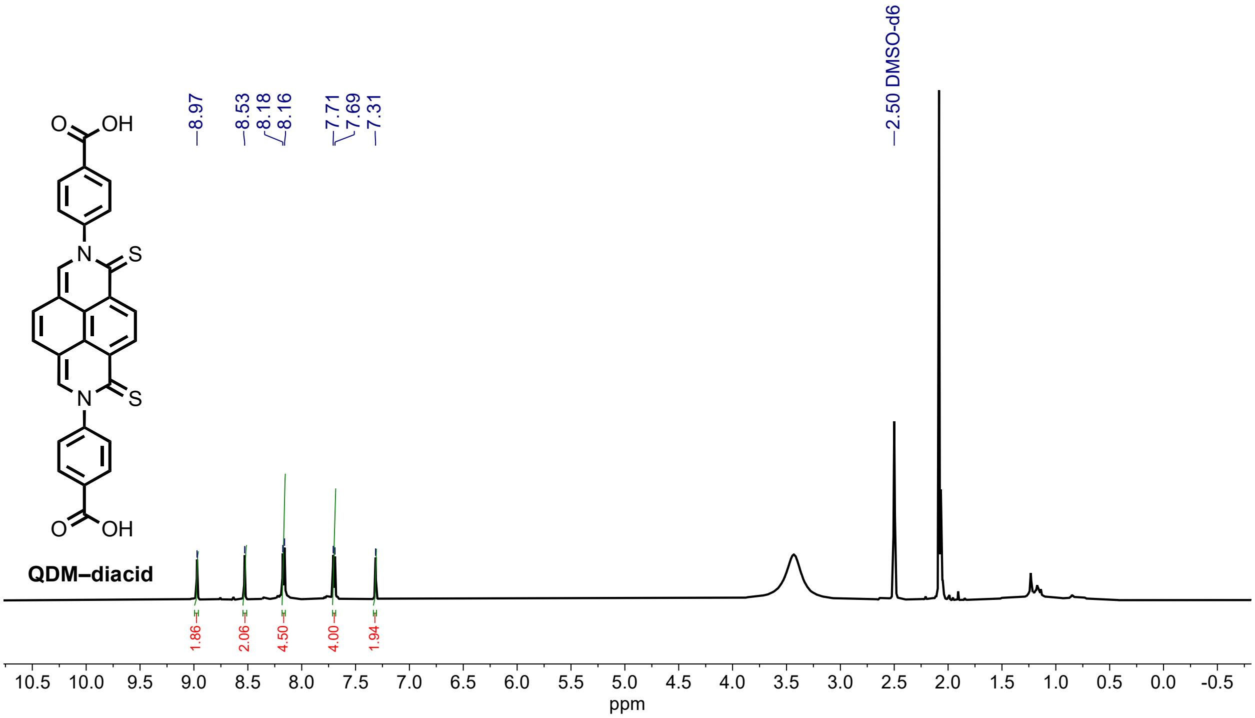

Figure S6. ^{1}H NMR (500 MHz) of QDM–diacid in DMSO-d_6.

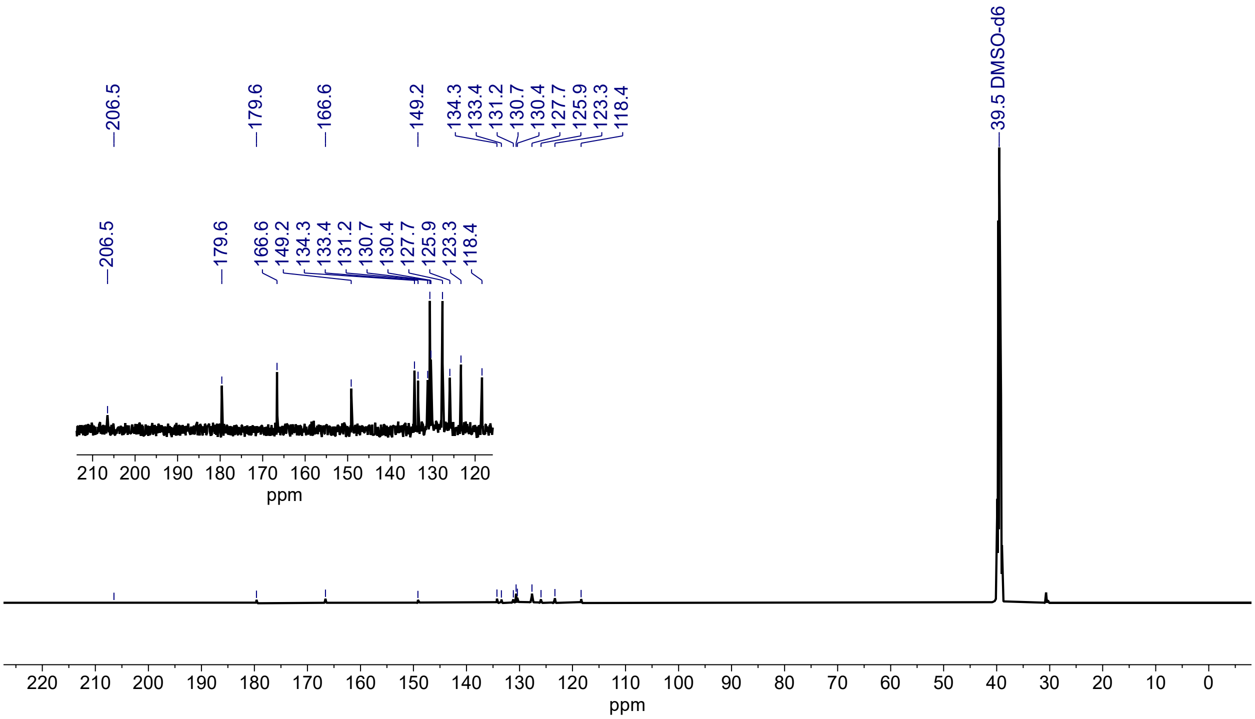

Figure S7. ^{13}C NMR (125 MHz) of QDM–diacid in DMSO-d_6.

SI-3. STM-BJ MEASUREMENTS

The single molecule conductance was determined following the same break-junction platform described above.

In the continue stretching measurement, a constant substrate bias of 200 mV was applied during the continue stretching measurement. The gold tip was first driven by a piezoelectric transducer (PZT) close to the QDM-diacid monolayer on the Au surface until the current reached a preset value, indicating the formation of molecular junctions. Subsequently, a current–distance trace was recorded while simultaneously retracting the STM tip. Approximately 2000 traces were collected for a given experimental condition at a retrace speed of 24nm/s to build the conductance histograms.

In the stretch-holding measurement, the molecular junction was held in position by retracting the STM tip to 1.5 nm. A rectangular modulation of 0.5 nm was then applied to the PZT. After each modulation, the junction is further stretched by 1nm and modulated again. This process is repeated to ensure clean formation and rupture of a fresh junction with an overall stretching of 15 nm. The junction modulation measurements were performed with the bias voltage 200mV applied to the substrate while the STM tip was grounded.

In the I–V measurement, the STM tip is driven toward the substrate to a preset current value under piezoelectric control. The feedback loop was then turned off, and an external voltage pulse was applied to the piezo to bring the STM tip 1.5 nm into contact with the substrate, ensuring stable electrical contact. Next, the tip was withdrawn until the current value reaches the single-molecule conductance value determined from the continuous stretching process. Once a single-molecule junction was established, the tip position was fixed to hold the QDM-diacid molecule between the electrodes, the bias will be swept from -1.1V to 1.1V (c to d in Figure S8a) to acquire the I–V characteristics (Figure S8b). A checking bias of -0.2 V was used only before (a to c in Figure S8a) and after (d to b in Figure S8a) the main sweep bias to verify junction stability and retention of the same single-molecule conductance state.

In the formation-probability analysis, for a given modulation amplitude or retraction speed, conductance traces showing a clear molecular plateau were selected for analysis. Each trace was assigned to the high conductance (G_H) or low conductance (G_L) state according to whether the plateau conductance fell within the corresponding conductance window defined from the 1D histogram. The formation probability of each state was then calculated as the number of traces assigned to that state divided by the total number of classified molecular traces under the same condition.

All conductance and I–V measurements were performed in air under ambient, non-electrochemical conditions using Au electrodes and freshly prepared samples. The observed conductance states were reproducibly obtained and reversibly interconverted during mechanical modulation. We therefore do not attribute the rectification to irreversible chemical decomposition such as oxidative decarboxylation, which is also chemically disfavored for the present aromatic, conjugated dicarboxylate scaffold under these conditions.

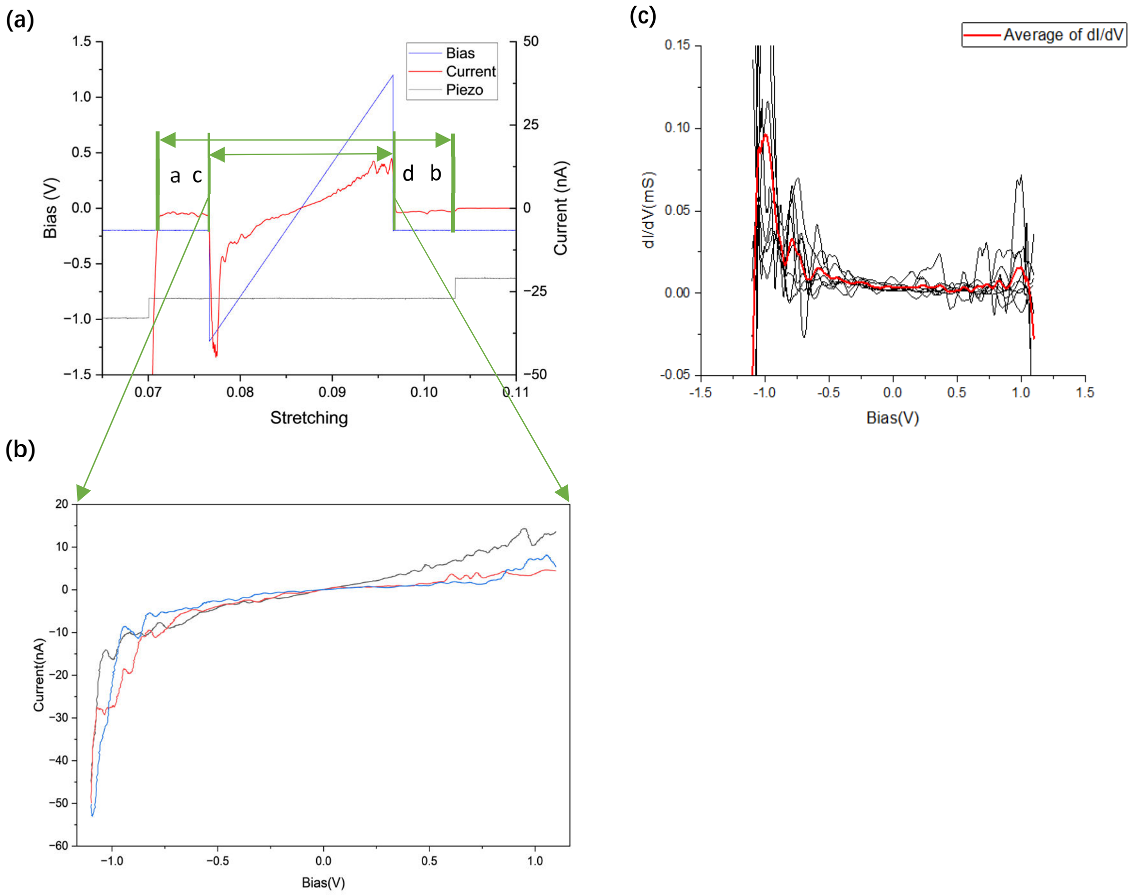

Figure S8. (a) Typical I-V curve for single molecule conductance with bias voltage sweeping during the stretch-holding process. (b) Individual I-V characteristics curves collected during the bias sweeping process. (c) Numerical differential conductance (dI/dV) spectra for QDM-diacid. Red line represents for average fitted dI/dV curve.

To further examine the rectification behavior, numerical differential conductance (dI/dV) spectra were constructed from the I–V data of QDM-diacid junctions (Figure S8c). The dI/dV profiles reflect the local density of states (LDOS) near the Fermi level within the applied bias range. The QDM-diacid molecule junction shows pronounced LDOS enhancement under negative bias, consistent with the higher current response in negative bias.

SI-4. THEORETICAL CALCULATION

Structure optimization along distance

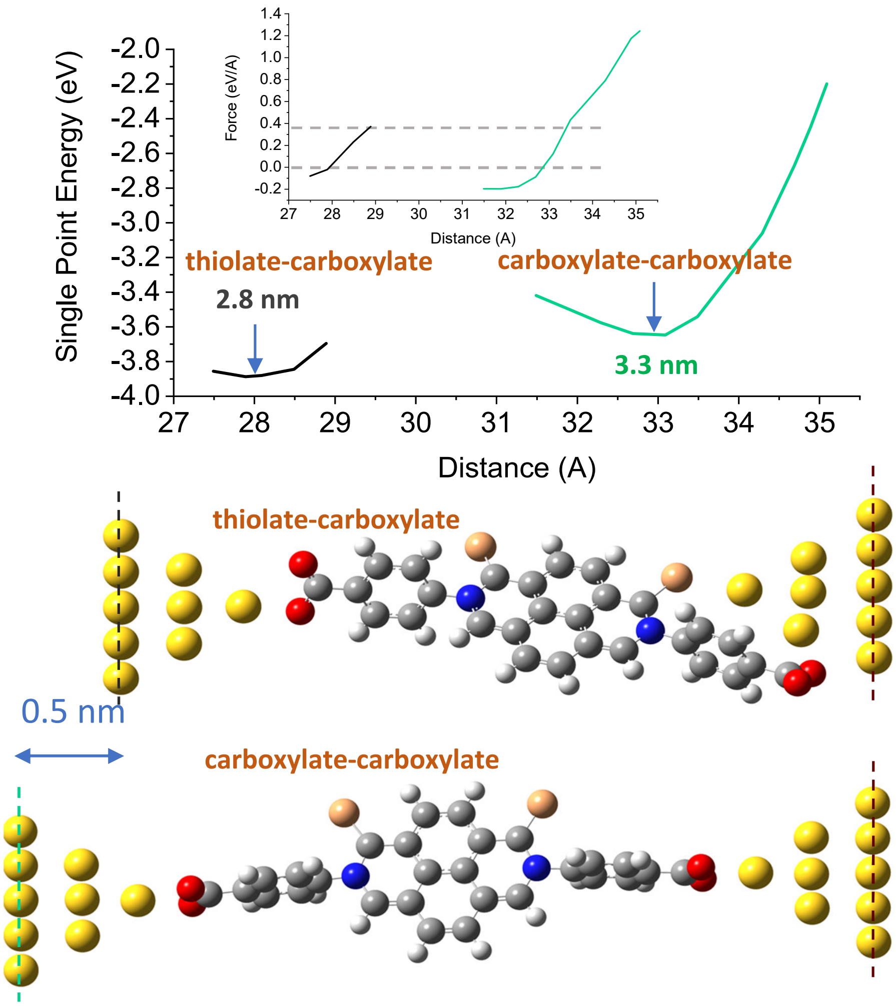

Figure S9. Single-point energies and corresponding structures of carboxylate-carboxylate and thiolate-carboxylate molecular junctions. The corresponding force curves are shown in the inset. The optimized equilibrium junction lengths are approximately 3.3 nm for the carboxylate-carboxylate configuration and 2.8 nm for the thiolate-carboxylate configuration.

To calculate the single-point energies distributed along the distance of the carboxylate-carboxylate and thiolate-carboxylate molecular junctions, we employed a three-layer pyramid-shaped gold tip structure as electrodes on both sides. The junction length was determined by measuring the distance between the final layers of the electrodes on both sides. We fixed the x and y coordinates of the outermost layers of gold atoms while freely optimizing its z coordinate and the three coordinates of the remaining atoms at the junction. This yielded the initial molecular junction lengths for each configuration. Subsequently, we fixed the x, y, and z coordinates of the outermost gold atoms and freely optimized the three coordinates of the remaining atoms at the

junction to obtain the single-point energy at specific distances. To maintain conformational consistency and obtain stretching/compression forces, each distance adjustment step was limited to ±0.4 Å. The atomic positions of the molecular junction obtained from the previous optimization step served as the initial positions for the next step, thereby generating the distance-energy curve for the molecular junction. (see Figure S9 and main text Figure 3b). Computational results indicate an optimized distance of 3.3 nm for the carboxylate-carboxylate junction and 2.8 nm for the thiolate-carboxylate junction. The corresponding force curves are shown in the inset. The results indicate that when the tensile force exceeds 0.4 eV/Å (approximately 0.64 nN), the thiolate-carboxylate molecular junction can elongate by about 0.15 nm, while the carboxylate-carboxylate molecular junction can elongate by approximately 0.25 nm. This indicates that the two configurations differ in their elongation plateau-length about 0.10 nm, which is consistent with the experimentally observed plateau-length difference. We optimized the molecular junction structures using the B3LYP functional and the LANL2DZ basis set in Gaussian 16[4] and obtained the single-point energy.

Transmission spectrum of the thiolate-carboxylate and carboxylate-carboxylate molecular junctions

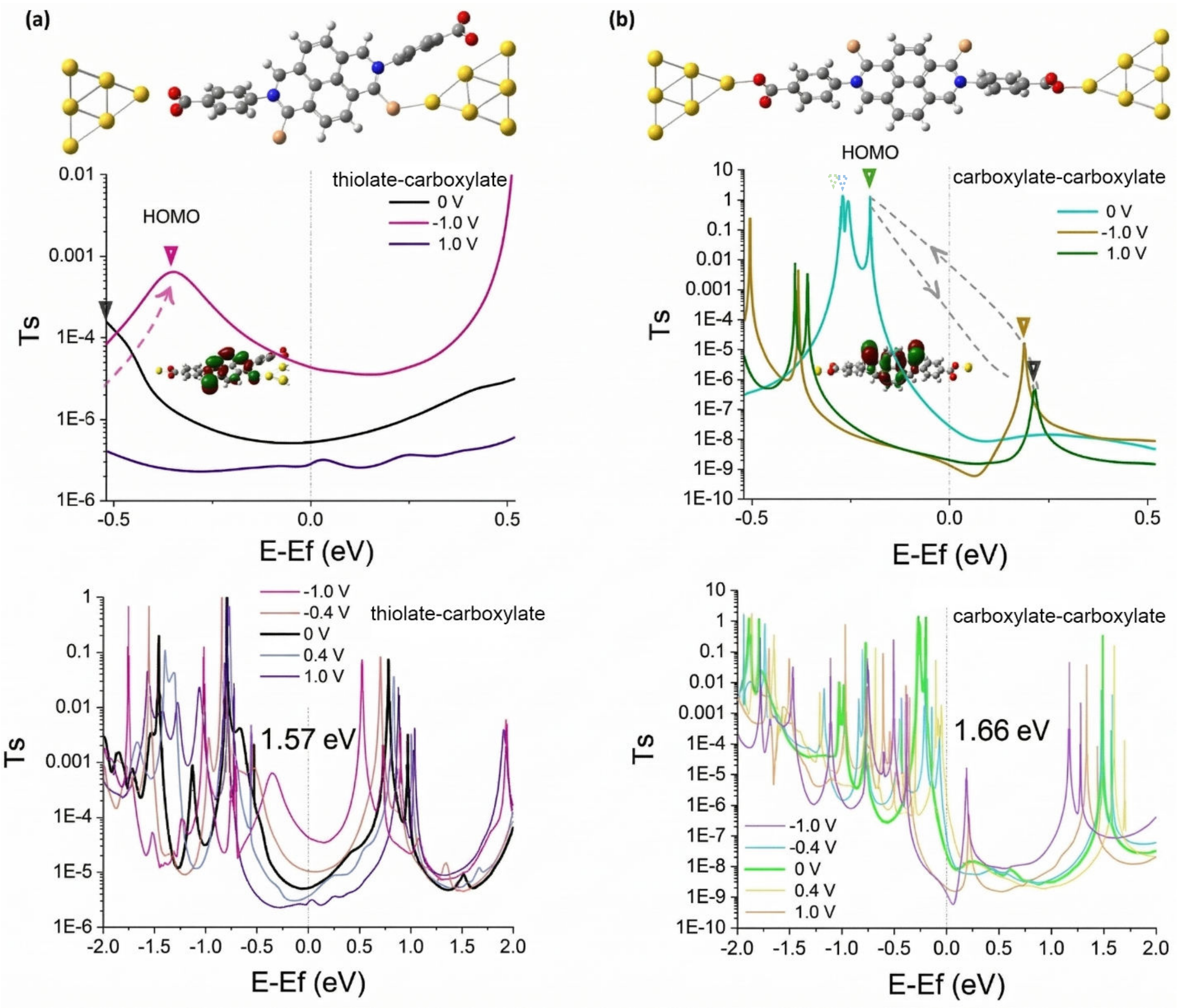

Figure S10. Transmission spectra of thiolate-carboxylate (a) and carboxylate-carboxylate (b) junctions and their corresponding structural configurations. The solid inverted triangles mark the positions of the highest occupied molecular orbital (HOMO) under different bias voltages. Green and blue dashed inverted triangles respectively mark the positions of HOMO-2 and HOMO-1 of carboxylate-carboxylate junction at zero bias. Dashed lines with arrows indicate the positional changes of HOMO from -1 V to 1 V. The inserts are the MPSH of HOMO. The HOMO-LUMO gap under 0 bias for thiolate-carboxylate is 1.57 eV and for carboxylate-carboxylate is 1.66 eV.

As the bias voltage changes from 1V to -1V, the highest occupied molecular orbital (HOMO) of the thiolate-carboxylate molecular junction monotonically shifts within the bias window and the HOMO-LUMO gap reduces, thereby inducing a rectifying effect in the device. From the electron distribution in the molecular orbitals, the electrons in the HOMO are primarily distributed near the S-Au contact, resulting in stronger coupling between this orbital and the right electrode. Consequently, the energy level of the HOMO can be monotonically controlled by the bias voltage. This leads to rectification in the molecular junction. For the carboxylate-carboxylate molecular junction, when the bias voltage changes from 1V to -1V, the energy difference between HOMO-

1 and HOMO gradually increases at higher voltages, while the HOMO-LUMO gap decreases. The local HOMO and the delocalized HOMO-1 undergo Fano resonance under negative bias. In the calculated transmission spectrum, this resonance is expressed as a broadened dip-like feature between HOMO and HOMO-1 rather than an ideal sharp peak-dip pair. (as seen in the -1.0V transmission spectrum). This induces rectification in the carboxylate-carboxylate molecular junction, though with a lower rectification ratio than the thiolate-carboxylate junction. The theoretical calculations agree with the experimental measurements.

The electron transmission spectra (Ts) of these molecular junctions are calculated using Non-equilibrium Green's function (NEGF) combined with density functional theory (DFT) method[5]. The 7×7 Au (111) periodic electrodes were used on both sides of the Au-Tip-molecule-Au-Tip junction. The exchange-correlation functional is generalized gradient approximation (GGA) with Perdew-Burke-Ernzerhof (PBE) formulation. Au atoms use single-ζ plus polarization (SZP) basis set, while the other atoms use double-ζ plus polarization (DZP) basis set. The real space grids use 300 Rydberg as a mesh cutoff. A 7×7 k-point grid was used for the Brillouin-zone (BZ) sampling in the transverse directions.

References

[1] J. Zhou, F. Chen, B. Xu, "Fabrication and Electronic Characterization of Single Molecular Junction Devices: A Comprehensive Approach", *J. Am. Chem. Soc.* **131**, (2009): 10439–10446.

[2] G. Yang, Y. J. Yun, F. Peccati, A. M. Jamhawi, N. Kamatham, S. Jockusch, G. Jiménez-Osés, A. J.-L. Ayitou, "Unraveling the Photophysical Characteristics, Aromaticity, and Stability of π–Extended Acene-Quinodimethyl Thioamides", *ChemPhysChem* **24**, (2023): e202200906.

[3] N. Kamatham, J. Li, S. Shokri, G. Yang, S. Jockusch, A. Y. Rogachev, A. J.-L. Ayitou, "Quinoidization of π-Expanded Aromatic Diimides: Photophysics, Aromaticity, and Stability of the Novel Quinoidal Acenes", *Eur. J. Org. Chem.* **2020**, (2020): 917–922.

[4] M. J. Frisch, G. W. Trucks, H. B. Schlegel, G. E. Scuseria, M. A. Robb, J. R. Cheeseman, G. Scalmani, V. Barone, G. A. Petersson, H. Nakatsuji, X. Li, M. Caricato, A. V. Marenich, J. Bloino, B. G. Janesko, R. Gomperts, B. Mennucci, H. P. Hratchian, J. V. Ortiz, A. F. Izmaylov, J. L. Sonnenberg, D. Williams, F. Ding, F. Lipparini, F. Egidi, J. Goings, B. Peng, A. Petrone, T. Henderson, D. Ranasinghe, V. G. Zakrzewski, J. Gao, N. Rega, G. Zheng, W. Liang, M. Hada, M. Ehara, K. Toyota, R. Fukuda, J. Hasegawa, M. Ishida, T. Nakajima, Y. Honda, O. Kitao, H. Nakai, T. Vreven, K. Throssell, J. A. Montgomery Jr., J. E. Peralta, F. Ogliaro, M. J. Bearpark, J. J. Heyd, E. N. Brothers, K. N. Kudin, V. N. Staroverov, T. A. Keith, R. Kobayashi, J. Normand, K.

Raghavachari, A. P. Rendell, J. C. Burant, S. S. Iyengar, J. Tomasi, M. Cossi, J. M. Millam, M. Klene, C. Adamo, R. Cammi, J. W. Ochterski, R. L. Martin, K. Morokuma, O. Farkas, J. B. Foresman, D. J. Fox, Gaussian 16, Rev. C.01, Gaussian, Inc., Wallingford, CT, 2016.

[5] M. Brandbyge, J.-L. Mozos, P. Ordejón, J. Taylor, K. Stokbro, “Density-functional method for nonequilibrium electron transport”, *Phys. Rev. B* **65**, (2002): 165401.
